# Acute effects of high-intensity interval training and moderate-intensity continuous training on executive functions in healthy older adults

**DOI:** 10.1038/s41598-025-91833-z

**Published:** 2025-02-25

**Authors:** Shirko Ahmadi, Mathieu Bélanger, Myles W. O’Brien, Pierre Philippe Wilson Registe, Olivier Dupuy, Said Mekari

**Affiliations:** 1 Vitalité Health Network, Dr. Georges-L.-Dumont University Hospital Centre, Moncton, NB Canada; 2 Department of Family Medicine, Université de Sherbrooke, Sherbrooke, Québec Canada; 3 Centre de formation médicale du Nouveau-Brunswick, Université de Sherbrooke, Moncton, NB Canada; 4 Department of Medicine, Université de Sherbrooke, Sherbrooke, Québec Canada; 5 Laboratory MOVE, Faculty of Sport Sciences, University of Poitiers, Poitiers, 6314 EA France

**Keywords:** Acute exercise, Older adults, Cognition, High intensity interval exercise, Moderate intensity continuous exercise, Ageing, Cognitive ageing

## Abstract

Numerous studies have demonstrated that executive functions benefit from high-intensity interval training (HIIT) and moderate-intensity continuous training (MICT). However, the immediate effects of HIIT and MICT on these functions in older adults have not been compared. This study aimed to examine the acute impact of HIIT and MICT on executive function components in this demographic. Twenty-five healthy community-dwelling older adults (15 females; average age 67.1 ± 4.5 years) participated. The study involved three sessions: an initial session with cognitive assessments (Stroop Task: Naming, Inhibition, and Switching) and a maximal continuous graded exercise test, followed by two sessions involving HIIT (15s at 100% peak power output, 15s rest, 2 × 20 min) or MICT (34 min at 60% peak power output) training protocols in random order. Cognitive tests were administered immediately after and 45 min post-training. The results showed a significant difference in Switching reaction times between MICT and HIIT, with HIIT showing a greater reduction in Switching times after 45 min (*p* = 0.019). In conclusion, our study indicates that HIIT’s beneficial effects on executive functions demonstrated a larger effect size than those of MICT. This suggests that brief, high-intensity exercise could be more effective in enhancing executive functions among older adults.

## Introduction

It is well established that regular physical activity is an effective strategy to prevent the cognitive decline associated with aging^1^. Numerous cross-sectional studies and interventional studies have shown that executive functions (EF), which represent higher order cognitive functions related to the management of emotions and attention, benefit from exercise training interventions^2^. Even a single bout of exercise can favorably impact EF^3^. Results from a literature review^4^and meta-analyses^5,6^consistently provide support for the positive effect of acute exercise on EF, especially among older adults. Many different ranges of exercise intensity have been reported to positively influence EF^5^, with effects lasting from 30 min up to two hours post exercise^7^. The distinctions across effects of various types of exercise sessions nevertheless remain unclear^8^.

High-intensity interval training (HIIT), characterised by brief durations of high intensity exercise interspersed with periods of low intensity exercise or rest, has been shown to improve cardiorespiratory fitness and vascular function to a greater extent than moderate-intensity continuous training (MICT) in older adults^9^. HIIT is generally well-tolerated, feasible, and may confer many health advantages to older adults^10^. A review by Ai et al.^11^ identified 57 acute outcomes of HIIT on EF across 24 articles. Overall, this review emphasized that among the EF sub-components: Inhibition, updating, and shifting, acute HIIT showed a trend toward improving the Inhibition component in younger adults. Despite the growing evidence for the benefits of exercise on EF, the comparative effects of HIIT and MICT on switching tasks in older adults—a critical aspect of cognitive flexibility—remain unclear. Understanding these effects is crucial, given the role of cognitive flexibility in maintaining functional independence in aging.

EF, which include inhibition, updating, and switching, are critical for managing attention and emotions. Among these, switching, or cognitive flexibility, is particularly relevant for older adults but has received less attention in acute exercise studies^12^. The role of acute HIIT on EF still needs to be investigated further in older adults, since only seven outcomes across the 24 articles regarding Switching were included. Further, a meta-analysis by Moreau & Chou^13^ reported that the small facilitating effect (d = 0.24) of HIIT on EF was only observed when compared with resting values (d = 0.34), but not when contrasted with MICT (d = 0.07) in younger adults. To our knowledge, only one paper has contrasted the effects of acute HIIT and MICT on cognition in older adults. Specifically, Tsai et al.^14^ reported that, in healthy older adults (*n* = 21), both HIIT and MICT improved post exercise reaction time on a delayed matching task (working memory). The acute effects of HIIT and MICT on Switching related tasks have not yet been compared in older adults.

In summary, whereas intensity of acute exercise sessions appears to modulate the effects of exercise on some components of EF in younger adults, more research is needed to directly compare the effects of HIIT and MICT on EF, particularly on Switching specific tasks in older adults^15^. Given the importance of such tasks in reflecting cognitive flexibility, which is crucial in aging, this study aims to fill this gap. Therefore, this cross-over study aimed to compare how each of HIIT and MICT can acutely influence EF components in older adults. Based on prior findings^14^, we hypothesize that both HIIT and MICT will improve EF components in older adults, with no significant differences between the protocols. By directly comparing the effects of HIIT and MICT on switching tasks, this study aims to advance our understanding of how acute exercise can optimize cognitive flexibility in aging populations, with potential implications for designing targeted interventions to prevent cognitive decline.

## Methods

### Participants

Twenty-five community-dwelling older adults (10 males, 15 females) were recruited to participate in the study from the Acadia Active Aging and Acadia Active for Life programs at Acadia University (see Table 1for participant characteristics). The sample size calculation was based on an expected effect size of 0.2 for the Switching reaction time. A calculated sample size of 25 was sufficient to achieve 80% power for an ANOVA analysis with repeated measures (3 time points), assuming an effect size of 0.2, a correlation of 0.7 among repeated measurements, and a 95% confidence level. All participants from these programs were recreationally active (Participating in aerobic activity up to twice a week for no more than 80 min at moderate intensity^16^). In this study, individuals aged 60 years and older are defined as ‘older adults’ for recruitment purposes. As part of the eligibility criteria, all participants were cognitively healthy, scoring at least 25 on the Mini-Mental State Examination, and had a normal-to-corrected vision. None of the participants had a history of neurological or psychiatric disorders, colour blindness, surgery with anaesthesia during the previous six months, nor did they have involuntary tremors, epilepsy, or drug/alcohol problems. Participants with cognitive, cardiovascular, metabolic, or musculoskeletal conditions that could affect their ability to exercise were excluded. All participants also completed the Get Active Questionnaire to screen for other contraindications for participation^17^. Participants were informed of the methods and study procedures verbally and in writing before providing written informed consent. All protocols and procedures conformed to the Declaration of Helsinki and were approved by the Acadia University Research Ethics Board (2021–3276).

**Table 1:** Descriptive statistics of study participants (*n* = 25).

Variables	Mean ± SD	(Minimum-Maximum)
Age (years)	67.1 ± 4.5	(60–76)
Height (cm)	167.9 ± 10.1	(153–190)
Weight (kg)	77.3 ± 13.9	(56–101.5)
HR (bpm)	70.0 ± 10.5	(52–92)
Systolic blood pressure at rest (mmHg)	128.7 ± 14.0	(107–156)
Diastolic blood pressure at rest (mmHg)	75.7 ± 8.4	(55–91)
V̇O_2max_ (ml/kg/min)	25.7 ± 6.6	(10.6–36.9)
Peak power (W)	151.7 ± 48.0	(65–245)

Note. HR: heart rate at rest; V̇O_2max_: maximal oxygen consumption; W: Watts.

### Experimental design

The study consisted of three sessions of approximately two hours, separated by at least 48 h but no more than one week. In order to minimize impact on cognitive functions, all sessions were performed at the same time of day. During the first session, participants completed a cognitive assessment (see below for cognitive assessment details) followed by a maximal continuous graded exercise test. The training protocols of the second and third sessions were assigned in a randomized counterbalanced order where participants completed a HIIT protocol or a MICT protocol. Cognitive tests were completed within five minutes of terminating the training protocol (T0) and again 45 min after completing their training protocol (T45). The training protocols for the second and third sessions were assigned in a randomized, counterbalanced order, ensuring that participants completed both the HIIT and MICT protocols. By having the same participants undergo both experimental conditions, counterbalancing was implemented to minimize the influence of potential confounding variables, thereby enhancing the internal validity of the study. Post-randomization, the groups were checked for balance across key variables (age, gender, baseline cognitive scores), confirming that they were similar. During the waiting period, participants were allowed to rest and drink water. Forty-five minutes after was selected as a secondary cognitive assessment interval considering many studies have observed prolonged effects up to 30 min post-exercise^18,19^ and fewer observing prolonged effects up to 60-minutes post exercise^20,21^. To minimize known confounding influences during exercise sessions, participants refrained from consuming caffeine or smoking and drinking alcohol within six hours of any testing, consistent with the exercise testing guidelines from the Canadian Society for Exercise Physiology^22^. Additionally consistent with the CSEP exercise testing guidelines, participants were asked to refrain from moderate (3–6 Metabolic Equivalent of Task (METs)) to vigorous (> 6 METs) exercise 24 h prior to their sessions and were also asked to consume a normal breakfast on the morning of the sessions. No effort was made to control the nutrition of the participants. All training was completed under the supervision of a trained exercise physiologist and visits were performed in a thermoneutral environment (21 ^◦^C).

### Cognitive testing protocol

Executive and speed of processing abilities were assessed with a computerized modified Stroop Task^23^. This test included three conditions. In the first condition (Naming), the participant had to read 1 of 4 possible words appearing on the screen: “RED”, “BLUE”, “YELLOW” or “GREEN”. These words were displayed in the same color as their meaning. The answers were mapped to the letters “u”, “i”, “o” and “p” on a keyboard, which participants used to give their answers with the right hand. The mapping remained the same throughout the task. The order was “index finger—red,” “middle finger—green,” “ring finger—blue”, and “little finger—yellow”. This condition is the simplest condition, and it provides a measure of processing speed. The second block consisted of a classic Inhibition task, which required naming the font color of a color-word, the meaning of the word being incongruent with the color of the font (e.g., the word “BLUE” displayed in green font). This condition is more difficult, as it requires participants to inhibit their reading of the color word and only respond to the color of the font. In these first three blocks, a fixation cross appeared for 500 ms, followed by the word for 3000 ms. The third block consisted of a Switching task, which was identical to the Inhibition task, except that for 25% of the trials a square appeared instead of the fixation cross. When the square appeared, participants were asked to read the colour-word, instead of naming the font color. The reading trials appeared randomly throughout the block, which makes this condition one of cognitive flexibility as the participant had to remember the rules (square vs. fixation cross) and respond based on these rules. Each of the three blocks contained 60 trials and the screen was blank between the trials. Before each condition, participants completed practice trials; 12 for the Naming condition, 12 for the Inhibition condition, and 20 for the Switching condition. During practice and experimental trials visual feedback (“Error”) was given for incorrect responses only. Reaction times to complete all trials for each condition were recorded as our primary outcome. Outliers in reaction times (defined as > 3 standard deviations from the mean) were removed from the analysis.

### Maximal continuous graded exercise test

The maximal continuous graded exercise test was performed on a cycle ergometer (Lode B.V., Groningen, The Netherlands). The initial workload was set at 1 W/kg body mass. The workload was increased by 15 W every minute until voluntary exhaustion. Strong verbal encouragement was given throughout the test. The power of the last completed stage was considered as the peak power output (PPO, measured in Watts). The relative volume rate of oxygen uptake (V̇O_2_, in mL/min/kg) was determined continuously on a 30-s basis using an automated cardiopulmonary exercise system (Parvo Medics TrueOne 2400, Salt Lake City, UT, USA). Gas analyzers were calibrated before each test using a gas mixture of known concentrations (15% O_2_ and 5% CO_2_). The primary criterion for attaining V̇O_2max_ was a plateau in V̇O_2_ (change < 2.1 mL/min/kg) despite an increase in workload. In the absence of a plateau, the attainment of V̇O_2peak_ was based upon a respiratory exchange ratio of ≥ 1.10 and the inability to maintain a pedaling cadence of 60 revolutions/min. Furthermore, V̇O_2max_ was the highest V̇O_2_ value attained during the test if the following criteria were observed: (1) a respiratory exchange ratio of ≥ 1.10 and (2) a peak heart rate ≥ 95% age-predicted maximum (i.e., 220 − age). Approximately 85% of the participants attained both criteria. Electrocardiographic activity was continuously monitored using a 12-lead electrocardiogram (ECG) (Philips, Netherlands). A certified exercise physiologist administered all tests.

### Acute exercise protocol

The HIIT session was based on previous studies that compared the time to exhaustion, participant preference, and time spent near the VO_2max_of various interval protocols^23,24^. The group performed 15 s cycling intervals at 100% PPO with 15 s of passive recovery (stop cycling, 0% of PPO). The cycling intervals were performed for two sets of 20 min (40 min total), with 5-minute of passive recovery between. The MICT protocol was based on the American College of Sports Medicine (ACSM) physical activity guidelines that recommend at least 30 min of daily moderate aerobic physical activity^25^. Continuous cycling at 60% PPO for 34 min was prescribed. This duration was implemented to ensure that the MICT protocol was isoenergetic to the HIIT protocol based on the assumption that mechanical efficiency, aerobic fitness, and PPO were similar between both exercise sessions; that 20 min at 100% PPO expends the same energy as 34 min at 60% PPO (MICT = 122 kJ vs. HIIT = 120 kJ for an individual with a PPO of 100 W). All exercise sessions began with a 5-minute warm-up and recovery at 25% PPO.

### Statistical analysis

Participants characteristics are described using descriptive statistics. After checking for normality via Shapiro-Wilk test, statistical analysis were performed using *t*-tests (when normality was confirmed) or Mann-Whitney U-tests (when data were non-normal) to verify whether the sequences of the two training methods (MICT before HIIT and HIIT before MICT) are associated with the three outcomes considered (Naming, Inhibition, and Switching) for the three time periods (Baseline, T0, and T45) by comparing the means for the two sequences for each of the three outcomes. The randomization was done to avoid any sequence effect. Therefore, this is done as a verification prior to further data analysis.

To examine if the exercises (HIIT and MICT) had an association with the three outcomes (Naming, Inhibition, and Switching), an analysis of variance (ANOVA) and a mixed-effects regression were conducted for a crossover study^26^, using five periods (Baseline, HIIT–T0, HIIT–T45, MICT–T0 and MICT–T45). On Stata, the command “pkcross” was used for the ANOVA, and with the design used for this study, the ANOVA for a crossover study was used to test the presence of four effects, which are: (1) the treatment effect, which corresponds to the direct effect of HIIT and MICT, (2) the period effect, which assess if the effect varies according to the period (T0 or T45) when it was assessed, (3) the potential carryover effect, which assesses whether the effect of one exercise protocol (HIIT or MICT) influences the subsequent protocol, and (4) the sequence effect to verify that the order in which types of exercise intensity were assigned does not influence results. The crossover analysis showed no significant carryover or sequence effects, suggesting that the order of exercise protocols did not affect the results. A mixed-effects regression was used to determine the size of each of the four possible effects described above. Results from the ANOVA for crossover analysis guided the choice of the random parameters to include in the regression analysis. When there is a period effect in the results of the ANOVA, a random effect for the period is included in the mixed-effects model. Various alternatives were tested, including period-by-treatment interaction, choosing only a random intercept, adding a random slope for the period effect, and the likelihood ratio test was used to retain the best-fitting model. The final mixed-effects model included a random intercept and random slope for the period effect. Paired *t*-tests were used as post-hoc analyses to provide more details in a visual representation of results on the effects identified during the mixed-effect regression and the ANOVA. Bonferroni correction was applied to the level of significance for those paired *t*-tests.

All statistical analyses were conducted using Stata version 17.0 and the significance level considered was set at *p* < 0.05 for the ANOVA and the mixed-effects regression analysis. For post-hoc paired *t*-tests, the significance level was set at *p* < 0.025 to adjust for multiple comparisons following Bonferroni correction.

## Results

### Participants

All 25 older adults (67.1 ± 4.5 years old) from the community successfully completed the required testing and were included in the final analysis. See Table 1 for participants characteristics.

After checking for normality and using the appropriate test, no sequence effects were noted across measures of reaction time for Naming, Inhibition, and Switching (*p* > 0.05). This verification confirms that the analysis could be run according to the planned crossover trial procedures.

### Naming results

**Fig. 1:**
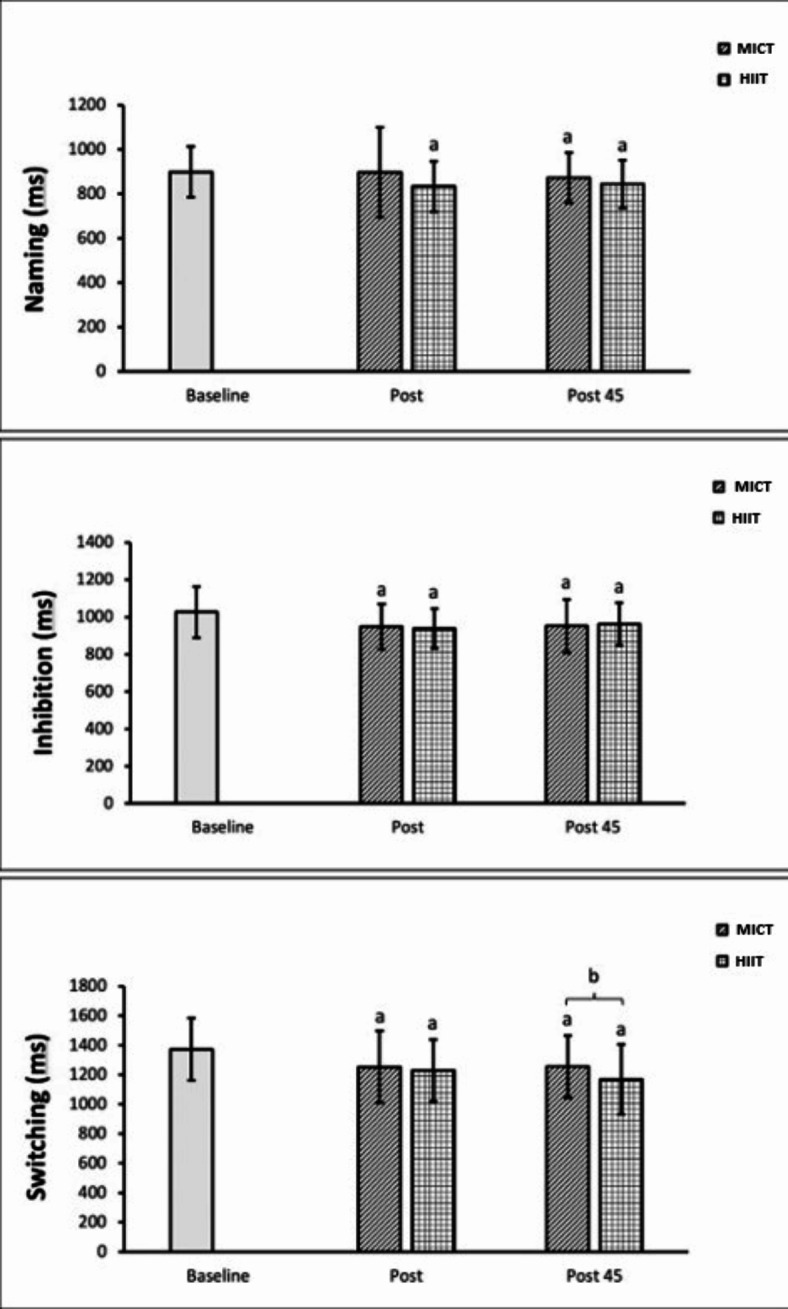
Post-hoc paired t-tests comparing variables. Error bars represent standard deviations of the mean. a: Indicates a significant difference between Baseline with Post and Post 45 measures. b: Indicates a significant difference between MICT and HIIT variables.

Using ANOVA results, significant variability was found within and between subjects’ measures of the Naming task (*p* = 0.041). This indicates a treatment effect was observed whereby exercise sessions (HIIT or/and MICT) significantly impacted “Naming”. Specifically, the mixed-effect regression model led to an estimate that the “Naming” task took an average of 884.8 ms at baseline. HIIT was associated with a significant reduction of 70.0 ms (CI [−126.8, −13.2]) in time to complete this task compared to baseline (Table 2). Post-hoc tests confirmed HIIT’s immediate post-training impact, reducing reaction time by 63.4 ms (*p* < 0.001), with a noTable 52.6 ms (*p* = 0.005) below baseline value after 45 min (Fig. 1).

**Table 2:** Mixed regression analysis for naming, Inhibition, and switching.

	Naming	Inhibition	Switching
	Coeff [CI]	Coeff [CI]	Coeff [CI]
**Sequence**			
HIIT-MICT vs. MICT-HIIT	30.1 [−63.8, 124.0]	11.7 [−80.7, 104.1]	93.5 [−59.5, 246.6]
**Treatment**			
HIIT MICT	−70.0* [−126.8, −13.2] −13.6 [−68.2, 40.9]	−77.6** [−123.4, −31.8] −68.7** [−112.7, −24.8]	−97.8* [−183.9, −11.7] −93.0* [−176.0, −10.0]
**Period**			
T0-1 T45-1 T0-2 T45-2	18.6 [−32.9, 70.1] −35.4 [−92.2, 21.4] 0 −25.8 [−80.3, 28.8]	−15.4 [−56.9, 26.2] −80.7** [−126.5, −35.0] 0 −66.0** [−110.3, −21.8]	−55.8 [−130.1, 18.4] −147.1** [−228.7, −65.4] 0 −91.5* [−180.9, −2.2]
**Carryover**			
HIIT MICT	−19.3 [−70.8, 32.2] 0	11.4 [−30.1, 52.9] 0	−67.2 [−140.9, 6.5] 0
**Constant**	884.8** [813.8, 955.8]	1020.5** [952.5, 1088.5]	1327.5** [1217.5, 1437.5]
**Random effects**			
Var(constant) Var(period) Var(residuals)	11199.5 [5764.1, 21760.1] --- 7495.9 [5559.9, 10106.1]	11367.3 [6040.5, 21391.4] 0 4864.4 [3608.1, 6558.0]	25740.6 [11770.3, 56292.4] 842.8 [224.5, 3163.8] 13964.1 [10047.8, 19406.7]

25 groups, 117 observations, *significant at 95%, **significant at 99%. Note: HIIT: high intensity interval exercise; MICT: moderate intensity continuous exercise.

### Inhibition results

The ANOVA analysis highlighted significant within- and between-participant variability. In particular, notable treatment effects were observed at the two post exercise measurement periods. The mixed-effect regression allowed to estimate that the mean reaction time associated to the Inhibition task was 1020.5 ms at baseline and both HIIT and MICT significantly led to a reduction in this measure (by 77.6 ms and 68.7 ms, for HIIT and MICT, respectively) immediately after exercise. This reduction persisted 45 min post-activity, with average declines of 80.7 ms and 66.0 ms in contrast with baseline for HIIT and MICT, respectively (Table 2). Post-hoc tests revealed no significant difference between HIIT and MICT in their immediate or 45-minute post-exercise effects on Inhibition reaction times (*p* = 0.228 and *p* = 0.634, respectively) (Fig. 1).

### Switching results

The ANOVA analysis highlighted significant within- and between-subjects variability, with substantial impacts from HIIT and MICT on Switching at different time points (immediately post-exercise and 45 min post-exercise). After adjusting for the design, the mixed-effect regression model estimated the mean baseline Switching at 1327.5 ms. HIIT reduced Switching reaction time by 97.8 ms immediately post-exercise, and this reduction was maintained at 147.1 ms after 45 min. MICT reduced Switching by 93.0 ms immediately post-exercise, with a smaller reduction of 91.5 ms after 45 min (Table 2). Post-hoc tests confirmed that HIIT and MICT effectively diminished reaction time for Switching after exercises and at 45 min post-exercise. Though initial results for MICT and HIIT were similar (*p* = 0.171), HIIT yielded a greater reduction in Switching (73.6 ms) than MICT after 45 min (*p* = 0.019) (Fig. 1).

## Discussion

Our study examined the impact of two different types of exercise protocols (HIIT and MICT) on the components of EF in healthy older adults. In line with our hypothesis, both protocols reduced reaction time, with the HIIT protocol leading to more substantial improvements in task-Switching-related activities. Specifically, we observed faster reaction times following acute exercise with the HIIT than MICT exercise protocol at T45. Although both exercise protocols demonstrated favorable immediate effects, HIIT presented a more sustained impact, resulting in a greater decrease in the Switching task performance time compared to MICT.

In the Naming task, both exercise protocols significantly improved reaction time, with HIIT showing a sustained effect even 45 min post-exercise. This finding supports the positive impact of acute HIIT on cognitive processing speed. This result is in line with the literature, since several studies using the Stroop paradigm have also observed the same effect of acute aerobic exercise on processing speed^27–29^. Both HIIT and MICT led to faster reaction times in the Inhibition task, with effects sustained 45 min post-exercise. However, no significant differences were found between the two protocols, suggesting that both are equally effective in enhancing inhibition control. This is consistent with findings of a meta-analysis indicating that both HIIT and MICT are equally effective at enhancing the Inhibition function among healthy individuals^30^. Given the unique benefits of each exercise method, individuals can select the one that aligns best with their personal needs and inclinations^30^.

The results of the Switching task were consistent with the Inhibition task. Both HIIT and MICT led to faster reaction times, with HIIT showing a significantly greater improvement 45 min post-exercise. Our findings are consistent with previous research in younger adults, which also shows that HIIT outperforms MICT in enhancing cognitive performance and flexibility^31,32^. As the first study comparing HIIT and MICT on the components of EF in older adults, our study corroborates with other studies’ findings in younger adults, again showing the superior impact of HIIT over MICT on cognitive performance and flexibility^18–21^. Advancing these findings from young healthy people to older adults is necessary to direct exercise training that attenuates or prevents the development of age-related cognitive impairments^33^. Our results suggest HIIT’s sustained and possibly superior impact on tasks requiring cognitive flexibility.

The deterioration of EF is an inevitable part of aging and can be a precursor to cognitive diseases^34^. Our study shows that a single HIIT session may temporarily enhance higher cognitive functions. This insight highlights the role of acute exercise in formulating chronic exercise regimens, which could be instrumental in slowing age-related cognitive decline. Considering previous research suggesting that HIIT can be safe and enjoyable for many healthy older adults^35,36^, we cautiously propose its inclusion in training programs aimed at enhancing or slowing cognitive decline. However, given individual differences in health status, physical capability, and potential risks, HIIT should be implemented with appropriate screening, supervision, and modifications as needed.

Our conclusions related to improvements in cognitive task Switching align with prior intervention studies involving healthy older adults^37,38^ and stroke patients^39^. Notably, these researchers found improvements in cognitive task Switching, supporting the theory that certain EF are more responsive to physical training and aerobic enhancements.

The cognitive advantages of exercise observed in our study can potentially be explained by the presence of brain-derived neurotrophic factor (BDNF), the increase in cerebral blood flow (CBF), and the release of lactate. Exercise, particularly HIIT, may enhance cognition through mechanisms such as increased BDNF^40,41^, enhanced CBF^42^, and the release of lactate^43^. These factors work together to support neural plasticity and improve cognitive function. In young subjects, it has been shown that the improvement in cognitive function in the Stroop test after physical exercise was correlated with blood lactate concentration, and the greater the intensity, the greater the release and the greater the cognitive improvement^44^. Especially as lactate interacts with the aforementioned BDNF. All mechanisms warrant more research.

The strength of this study lies in its standardization of energy expenditure across both MICT and HIIT protocols. This approach ensures a fair comparison by removing energy usage as a confounding factor. The crossover design strengthens the study by minimizing individual variability, allowing participants to serve as their own controls. This enhances the statistical power and internal validity of our results. This feature consequently aids in detecting actual effects. Such aspects considerably enhance the study’s validity, reliability, and practicality, marking it as an important contribution to research. Nevertheless, the study design does not allow to establish mechanistic underpinnings of our observations. A limitation of the study is that it was conducted on cognitively healthy adults, so the applicability of the findings to those with cognitive impairments is uncertain. Additionally, energy expenditure during the rest intervals of the HIIT protocol was not measured, which could impact the accuracy of the total energy expenditure comparison. We also recognise that baseline measurements not performed just prior to exercise may be considered a limitation as well. Therefore, more expansive research that includes a more representative sample and investigates the underlying mechanisms to ensure robust and generalizable results is needed.

## Conclusion

In conclusion, both HIIT and MICT positively impact executive function, with HIIT demonstrating more sustained effects, particularly on cognitive flexibility. These findings suggest that higher-intensity exercise may be more effective in improving EF in older adults, offering potential benefits for cognitive health interventions.

## Data Availability

The datasets generated during and/or analyzed during the current study are not publicly available, but are available from the corresponding author via email upon reasonable request.
